# A Novel Intelligent Fault Diagnosis Method for Bearings with Multi-Source Data and Improved GASA

**DOI:** 10.3390/s24165285

**Published:** 2024-08-15

**Authors:** Qingming Hu, Xinjie Fu, Yanqi Guan, Qingtao Wu, Shang Liu

**Affiliations:** 1 School of Mechanical and Electrical Engineering, Qiqihar University, Qiqihar 161006, China; xinjiefu2022@163.com (X.F.); 03312@qqhru.edu.cn (Y.G.); 305deshenghuo@163.com (Q.W.); shangliu2009@126.com (S.L.); 2 The Engineering Technology Research Center for Precision Manufacturing Equipment and Industrial Perception of Heilongjiang Province, Qiqihar University, Qiqihar 161006, China; 3 The Collaborative Innovation Center for Intelligent Manufacturing Equipment Industrialization, Qiqihar University, Qiqihar 161006, China

**Keywords:** fault diagnosis, rolling bearing, deep learning, multi-source data, genetic algorithm, simulated annealing algorithm

## Abstract

In recent years, single-source-data-based deep learning methods have made considerable strides in the field of fault diagnosis. Nevertheless, the extraction of useful information from multi-source data remains a challenge. In this paper, we propose a novel approach called the Genetic Simulated Annealing Optimization (GASA) method with a multi-source data convolutional neural network (MSCNN) for the fault diagnosis of rolling bearing. This method aims to identify bearing faults more accurately and make full use of multi-source data. Initially, the bearing vibration signal is transformed into a time–frequency graph using the continuous wavelet transform (CWT) and the signal is integrated with the motor current signal and fed into the network model. Then, a GASA-MSCNN fault diagnosis method is established to better capture the crucial information within the signal and identify various bearing health conditions. Finally, a rolling bearing dataset under different noisy environments is employed to validate the robustness of the proposed model. The experimental results demonstrate that the proposed method is capable of accurately identifying various types of rolling bearing faults, with an accuracy rate reaching up to 98% or higher even in variable noise environments. The experiments reveal that the new method significantly improves fault detection accuracy.

## 1. Introduction

With the increasing demands for the complexity and precision of modern machinery, its normal operation and safety become important assessment factors [1]. Rolling bearings, as integral components of rotating machinery [2], are extensively utilized in numerous significant industrial sectors, including wind power generation, aircraft engines, high-speed train wheels, and other crucial fields. Consequently, machine fault detection and diagnosis have received significant attention in the realm of industry [3].

In recent years, the fault diagnosis techniques have made significant progress [4]. A considerable number of academics have developed a multitude of effective fault diagnosis methodologies. For example, Gao et al. [5] proposed a fault diagnosis method for rolling bearings based on least squares support vector machine (LS-SVM). They utilized particle swarm optimization and 10-fold cross-validation to optimize the model parameters, achieving accurate classification and fast diagnosis of compound faults. Pu et al. [6] introduced restricted sparse networks (RSNs) with a high-power feature extraction module for efficiently extracting radial basis function features (RBFFs), demonstrating fault diagnosis accuracy comparable to the state-of-the-art methods. Combining the HHL algorithm in quantum computing with LS-SVM, Li et al. [7] proposed a quantum least squares support vector machine (QSVM) for fault diagnosis. Lei et al. [8] combined the Markov transition field (MTF) with a multi-scale feature aggregation convolutional neural network (MFACNN) to tackle issues of excessive parameters, slow training speed, and insufficient generalization in the traditional CNNs.

While the aforementioned methodologies have achieved considerable advancements, the diagnostic reliability is constrained by the inability of single-source data to provide sufficient fault information [9]. It is shown that the vibration signals collected by a single sensor can no longer meet the diagnostic requirements of complex systems [10]. Additionally, the data from a single sensor are often incomplete, leading to low diagnostic accuracy in complex scenarios. Multi-source data fusion is an effective method for fault diagnosis since the signals are usually complementary [11]. Full utilization of the sensors installed at various locations can enhance the completeness and performance of diagnosis models [12]. Thus, integrating data from different sources can improve the accuracy and reliability of fault diagnosis compared to using a single sensor. Wu et al. [13] collected homologous information from numerous sensors and fused the data in the spatial and time domains, validating the correctness and effectiveness of their method using the fault test signals of planetary gearboxes. Tong et al. [14] proposed a multi-sensor information fusion framework with coordinated attention mechanisms to achieve the fault diagnosis of rolling bearings. Liu et al. [15] introduced a novel multi-sensor information fusion framework and a multi-sensor-based frequency information fusion method to classify multi-frequency features. Wang et al. [16] adopted a temporal–spatial graph neural network with an attention-aware module to achieve multi-source information fusion, demonstrating effectiveness and robustness in bearing fault diagnosis.

Despite the fact that deep learning techniques are emerging as a promising solution, practical engineering applications still face challenges in determining reliable and effective hyperparameters. Inappropriate hyperparameter settings can significantly impair the CNN fault diagnosis capabilities. To address this, some scholars have combined intelligent optimization algorithms (e.g., particle swarm optimization [17] (PSO), the genetic algorithm [18] (GA), or simulated annealing [19] (SA)) with CNNs for improved results. Liu et al. [20] proposed a bearing fault model based on a PSO-fused CNN, which adaptively adjusts the hyperparameters of the model through PSO. Chen et al. [21] applied Quantum Particle Swarm Optimization to increase the richness of the particles and make it easier to find the global optimal solution of an adaptive CNN. Rajagopalan [22] focused on diagnosing multi-class mass imbalance faults using a genetically optimized 1D-CNN, achieving a fault prediction accuracy of 97.47%. He et al. [23] proposed a bearing fault diagnosis method that combines wavelet packet transform with a CNN optimized by simulated annealing. Bai et al. [24] utilized the global optimization capability of a genetic algorithm to enable the autonomous evolution of the CNN. The method was validated using the measured signals from a planetary gearbox, resulting in higher fault diagnosis accuracy.

Meanwhile, due to the inherent limitations of the GA, it is susceptible to premature convergence [25]. This study integrates the local search capability of the SA into the GA optimization process to enhance the CNN performance and improve the diagnostic accuracy.

Firstly, multi-source data are collected and preprocessed. The datasets are then divided into training, validation, and test sets with a ratio of 0.5, 0.25, and 0.25, respectively.

Secondly, the GASA-MSCNN fault diagnosis method is proposed. This method utilizes GASA to determine the hyperparameters of an MSCNN for rolling bearing fault diagnosis.

Finally, the method’s validity and practicality are verified using actual data from Paderborn University.

The rest of this paper is structured as follows: Section 2 elucidates the theoretical basis. Section 3 provides a comprehensive introduction to the proposed method and the detailed implementation process of fault diagnosis. Section 4 presents the experimental verification and analysis. Section 5 summarizes the article.

## 2. Theoretical Background 

### 2.1. Convolutional Neural Networks

**Figure 1:**
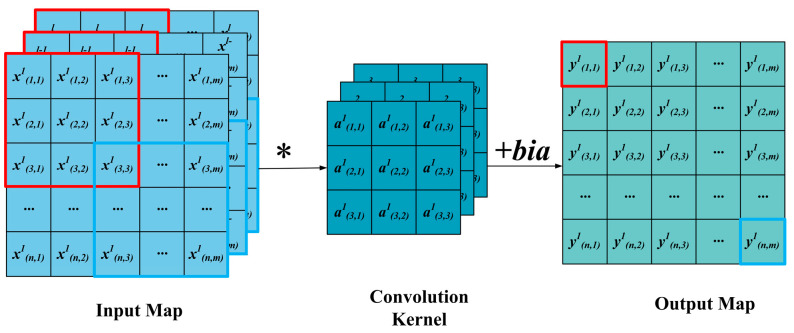
Schematic of CNN.

As a type of machine learning, convolutional neural networks (CNNs) are inspired by the neurons in the visual nervous system [26]. They play a pivotal role in the domain of deep learning algorithms. As feedforward neural networks, CNNs can extract features efficiently and reduce the computational load of the network model through local weight sharing and sparse connectivity [27]. A typical CNN structure is composed of a convolution layer, a pooling layer, and a fully connected layer. Among them, the convolution and pooling layer are typically connected in an alternating manner, and the fully connected layer is composed of several multi-layer perceptrons [28]. During the operation of the convolution kernels, they scan the input features and sum them by multiplying the matrix elements and adding the bias within the receptive field. The operation of the convolution layer is illustrated in Figure 1, and the mathematical description is provided as follows:

(1) $$y_{n}^{l}=f\left(\sum_{c=1}^{C}x_{c}^{l-1}\times a_{n,c}^{l}+b\right)$$

where $y_{n}^{l}$ is the *n* th feature map of the *l* th convolutional layer, $x_{c}^{l-1}$ denotes the input feature, $a_{n,c}^{l}$ represents the *n* th convolution kernel, *f*(·) is the activation function, and *C* stands for the number of input channels.

**Figure 2:**
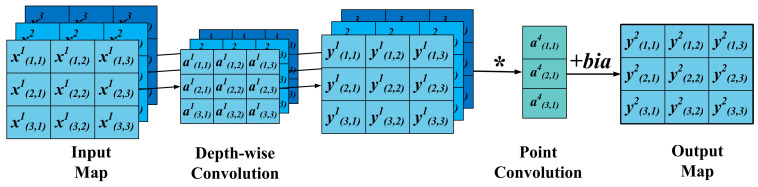
Schematic of DWSC.

As a variant of CNN, depth-wise separable convolutions (DWSCs) include depth convolution and point convolution [29], which can reduce the computational complexity and space complexity of each convolutional layer. The workflow of DWSC is shown in Figure 2, and the output can be calculated as follows:

(2) $$y_{c}^{l}=f\left(x_{c}^{l-1}\times a_{c}^{l}+b\right)$$

(3) $$z_{n}^{l}=f\left(\sum_{c=1}^{C}p_{n}\times y_{c}^{l}+b\right)$$

In the formula, $y_{c}^{l}$ and $z_{n}^{l}$ are the depth convolution and the point convolution, respectively.

**Figure 3:**
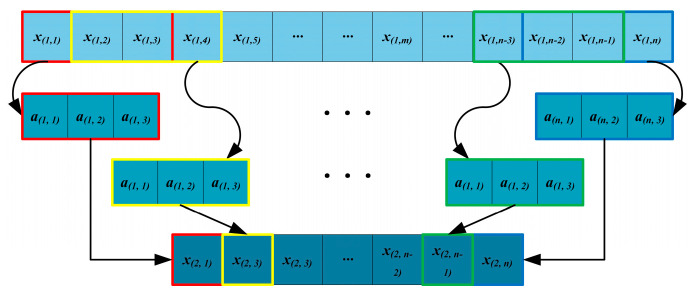
Schematic of 1D − CNN.

For a one-dimensional input, the signals are convolved by sliding a filter, performing element-wise multiplication, and then summing all elements to obtain the value at the corresponding position in the output feature map. The specific representation process is illustrated in Figure 3.

### 2.2. Genetic Algorithm

Genetic algorithms (GAs) are computational models inspired by natural evolutionary systems, proposed in the 1870s by John Henry. As a type of bio-inspired algorithm, their primary objective is to optimize complex parameter selection tasks [30]. These models employ theories of biological evolution and Darwin’s survival of the fittest. The algorithm starts by initializing the population, setting its scale, and generating an initial population. Subsequently, continuous selection, crossover, and mutation processes evolve the population, forming new generations.

During the selection process, the GA needs to determine the probability of each individual being selected. Roulette wheel selection [31] is a strategy that selects individuals based on their fitness ratio. The probability of selection is expressed as

(4) $$I_{n}=\frac{k}{F_{i}}$$

(5) $$P_{n}=\frac{I_{n}}{\sum_{n=1}^{N}I_{n}},n=1,2,\cdots ,N,$$

where *I_n_* denotes the reciprocal of individual fitness, *F_i_* is the fitness value of the *i* th individual, *k* represents the coefficient, and *N* stands for the population scale.

The cross operation is a common method of gene manipulation. It involves transferring the superior genes of the previous generation to the next, followed by a random cross to create a new optimization space. Mutation manipulation is a common method of genetic manipulation employed to introduce novel genetic alterations, thereby expanding the diversity of the search space. Meanwhile, it is necessary to replace unsuitable candidates based on the fitness function. The process persists until a predefined stopping condition is met. The equation for the fitness function can be illustrated as follows:

(6) $$F=k[\sum_{i=1}^{n}abs(t_{i}-y_{i})]$$

In which *n* indicates the number of output nodes, *t_i_* represents the actual value of the *i* th node, *y_i_* denotes the *i* th node predicted value, and *k* is the coefficient.

**Figure 4:**
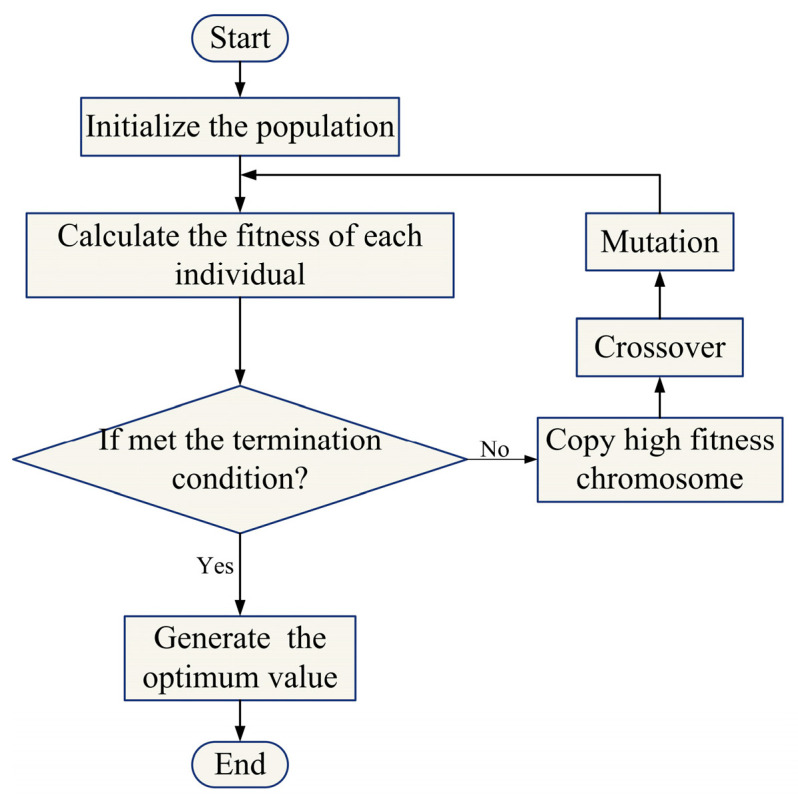
Flowchart of genetic algorithm.

The specific procedures of the GA are summarized in Figure 4.

### 2.3. Simulated Annealing Algorithm

The simulated annealing (SA) algorithm was proposed by Metropolis et al. in the 1950s. The fundamental principle of SA is based on the metallurgical annealing process in physics [32]. Initially, the metal material is heated to a specific temperature to melt it. Afterward, the material is cooled into a solid, forming a regular microstructure to minimize internal energy. When heated, the particles inside the solid continuously accelerate and move non-uniformly due to the increase in temperature, resulting in an increased internal energy. As the temperature decreases, they decelerate and tend to become ordered, reaching equilibrium at a specific temperature. Once ambient temperature is reached, the particles attain their lowest internal energy state and achieve thermal equilibrium. The fundamental principle of the SA algorithm is that it can accept a solution inferior to the current one with a certain probability P, in accordance with the Metropolis sampling rule [33]. This enables it to escape from a local optimal solution and to identify the optimal solution amongst all possible solutions. The cooling equation can be expressed as follows:

(7) $$T(t)=\frac{T_{0}}{\mathrm{lg}(1+t)}$$

where *T*(*t*) is the temperature value at time t, and *T*_0_ denotes the high temperature.

**Figure 5:**
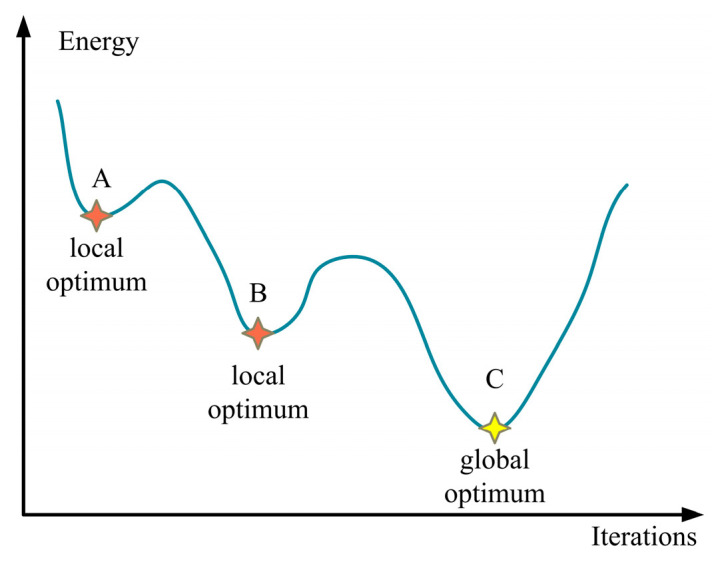
Schematic diagram of simulated annealing algorithm.

Figure 5 shows the schematic diagram of the simulated annealing algorithm.

One advantage of SA is its capacity to escape local optima, thereby demonstrating high efficacy and global search capabilities for complex optimization problems. According to Figure 5, as the number of iterations increases, the SA algorithm initially identifies the local optimum at point A. It then continues to calculate, discovering that point B has a lower energy value and updating the optimal solution to point B. Iteratively, the algorithm finds that point C has even lower energy than point B. With no lower energy found in subsequent iterations, point C is identified as the global optimum.

## 3. The Proposed Method

### 3.1. GASA

**Figure 6:**
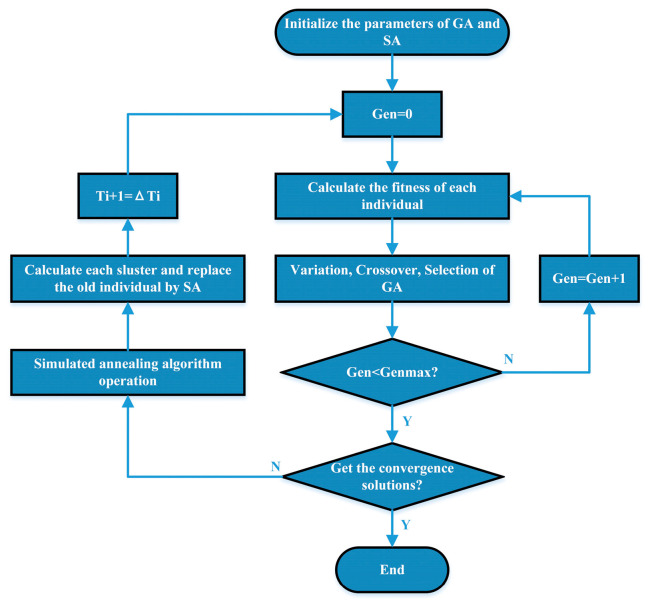
Flowchart of the proposed optimization algorithm based on GASA.

Extensive research has revealed certain disadvantages of GA, such as lower efficiency compared to other optimization algorithms and susceptibility to premature convergence [18]. SA also has some disadvantages, such as taking a long time to reach the optimization result [33]. Additionally, SA parameters are difficult to adjust for specific problems. To address these shortcomings, we propose a novel method that combines GA and SA to optimize convolutional neural networks (CNNs). GASA is a comprehensive method that combines the advantages of both GA and SA while mitigating their disadvantages. It not only has the global search ability of GA but also the local optimization ability of SA. In this paper, we use it to optimize the hyperparameters of CNNs. The first aspect is to initialize the population and calculate the fitness of each operator. Then, the crossover and mutation operators are set, and the fitness of all operators in the subgroup is ordered from largest to smallest. After that, the GA performs a global search to generate new populations. It then calculates the fitness value of the new population and replaces the old population by using SA. Finally, the result is output according to whether the convergence condition is achieved. Figure 6 shows the flow of the proposed method. In this study, the initial temperature of the annealing algorithm is 100, the decline rate is 0.95, the population size of the genetic algorithm is 10, the maximum genetic generation is 50, and the crossover and mutation probabilities are 0.5 and 0.2, respectively.

### 3.2. The Structure of the Model

**Figure 7:**
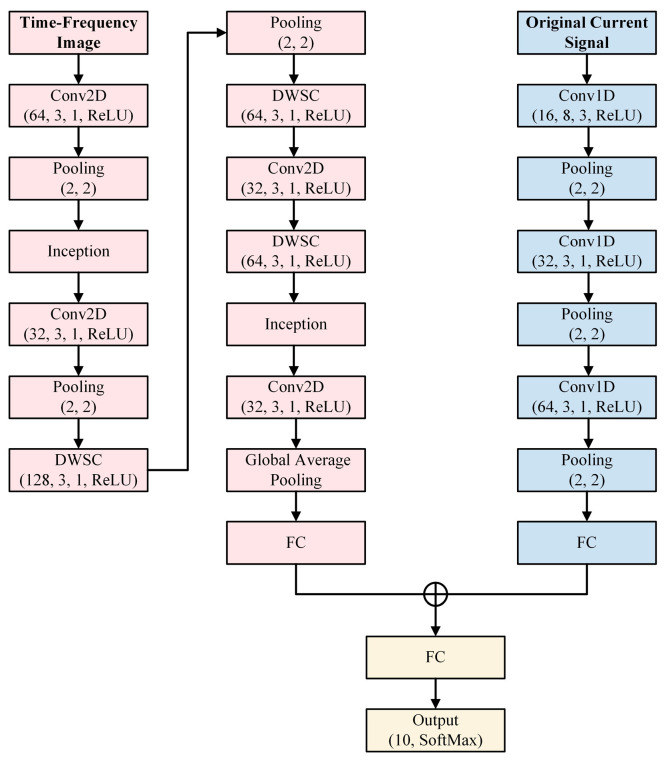
The structure of the model.

All experiments are performed on a PC with Windows 10 operating system, Intel Core(TM) i7-10700F CPU @ 2.90 GHz, and 64 GB RAM, NVIDIA GeForce RTX 2070 SUPER as the graphics card. Of particular note, we use MATLAB 2022a to obtain CWT time–frequency images, and all the models are implemented by Python 3.8 environment in Keras framework using TensorFlow as a backend. The fault diagnosis model proposed in this paper employs time–frequency images and original motor current signals as inputs to realize different types of fault diagnosis. In the MSCNN model, low-level fault features are extracted by the low-level convolution layer, while the abstract feature of the fault type is extracted by the high-level convolution layer. Taking Conv2D (64, 3, 1, ReLU) in the model as an example, Conv2D represents a two-dimensional convolution, where (64, 3, 1, ReLU) specifies the convolution kernel parameters and activation functions. The channel of the convolution kernels, the sizes of the convolution kernels, and strides in the convolutional layer are 32, 3 × 3, and 1, respectively. During the MSCNN model’s pretraining, the Adam optimizer is used for network training. This process allows the cross-entropy loss function and backpropagation to continuously optimize and adjust the model’s weight parameters, ultimately obtaining the optimal parameters for data feature extraction. The detailed structure of the proposed multi-source data convolutional neural network (MSCNN) is shown in Figure 7. 

**Figure 8:**
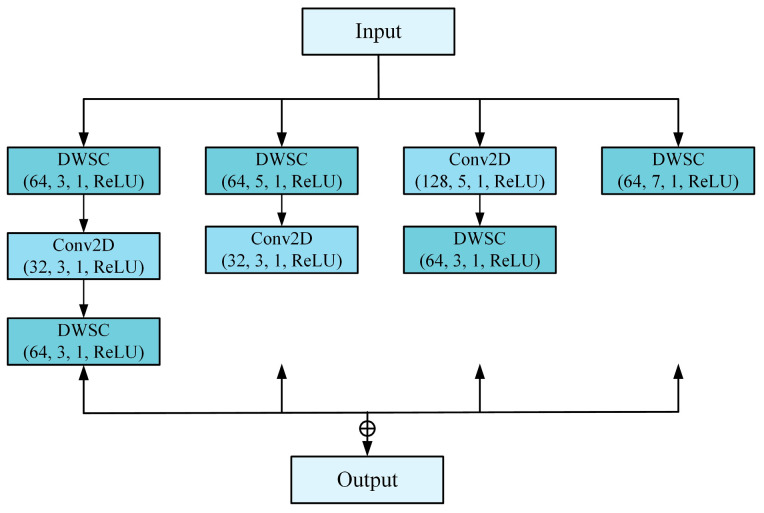
Schematic diagram of improved Inception layer.

The network branch that extracts features from two-dimensional time–frequency graphs consists of 4 convolutional layers, 3 depth-wise separable convolutional layers, 3 inception layers, and 1 fully connected layer. The network branch that employs one-dimensional current signals for feature extraction comprises 3 convolutional layers and 1 fully connected layer. The data from these two fully connected layers are then combined into a new fully connected layer for classification. Figure 8 indicates Inception structure in this study, which has been slightly improved.

In this structure, both depth-separable convolution and standard convolution are used. The sizes of the kernels in the four channels are three consecutive 3 × 3 kernels, two consecutive 5 × 5 and 3 × 3 kernels, and one 7 × 7 kernel. The softmax function is connected in the model output layer to achieve classification.

### 3.3. The Fault Diagnosis Process Using GASA-MSCNN

**Figure 9:**
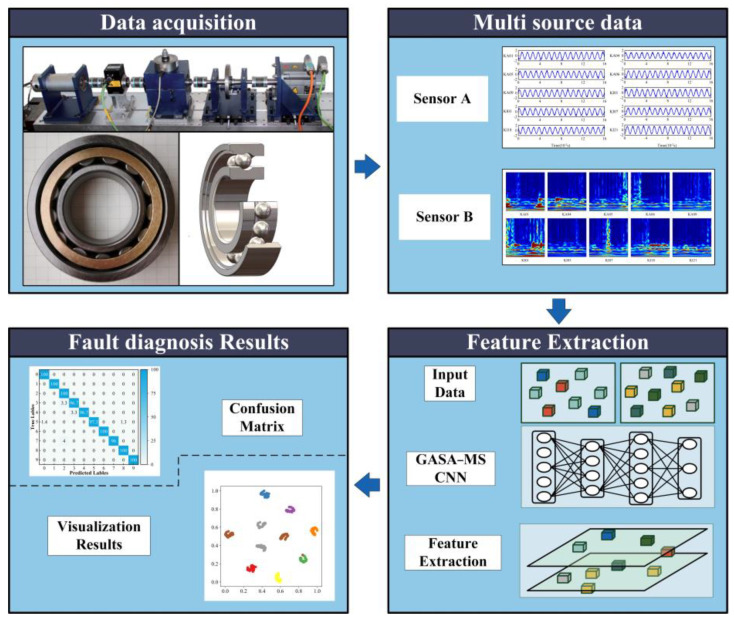
Overall framework of the proposed method.

To fuse multi-source data for fault diagnosis of rolling bearings, this paper proposes a novel intelligent fault diagnosis method for bearings with multi-source data and improved GASA. When the time–frequency graph of the vibration signal is used as input, the two-dimensional convolution is used to extract features. It is noted that we combine the multi-scale feature extraction and DWSC for better fault diagnosis, which can not only maintain feature extraction capability but also reduce the number of parameters. When the motor current signal is used as input, one-dimensional convolution is used for feature extraction to achieve better fault diagnosis results [34]. The flowchart of bearing fault diagnosis with multi-source data and improved GASA is present in Figure 9. The process is divided into the following steps.

Step 1: Collect vibration and current signals under various conditions through a data acquisition system.

Step 2: Multi-source data are preprocessed, and the datasets are partitioned into training, validation, and test sets.

Step 3: GASA is utilized to optimize the hyperparameters of the model, and the optimization results are applied to the model for fault diagnosis. 

Step 4: Diagnose the test dataset by previously saved model and evaluate the precision of the fault diagnosis.

## 4. Experimental Validation

### 4.1. Dataset Description

**Figure 10:**
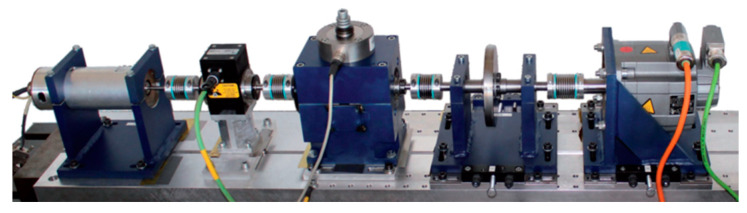
The experimental test rig of PU dataset test rig.

The experiment is based on the Paderborn University (PU) bearing dataset provided by the Paderborn Bearing Data Center [35]. The PU dataset includes both human-made bearing damage and actual damage from accelerated life experiments. A piezoelectric accelerometer is used to measure the vibration signals with a sampling frequency of 64 kHz, and the motor current signals are digitized and saved synchronously. The test rig of the PU datasets is displayed in Figure 10.

In the PU dataset, four distinct operational scenarios are presented, each achieved by modifying the velocity of the drive system, the radial force exerted on the test bearing, and the load torque on the drive system. This allows the test bearing to function under varied operational conditions. The parameters are shown in Table 1. To explore whether the model has satisfactory diagnostic capacity in the case of real damage, different fault types of bearings were selected. The details are shown in Table 2.

**Figure 11:**
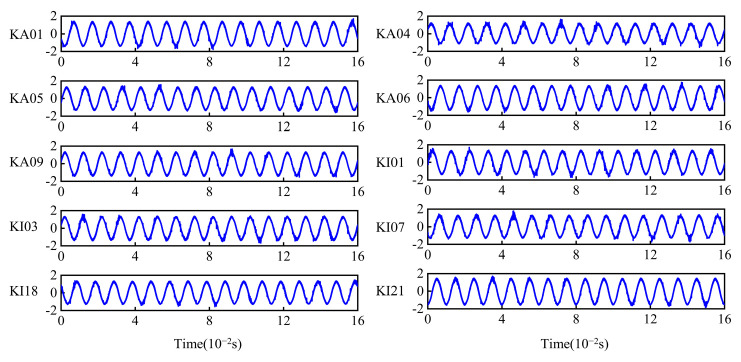
The motor current signals.

**Figure 12:**
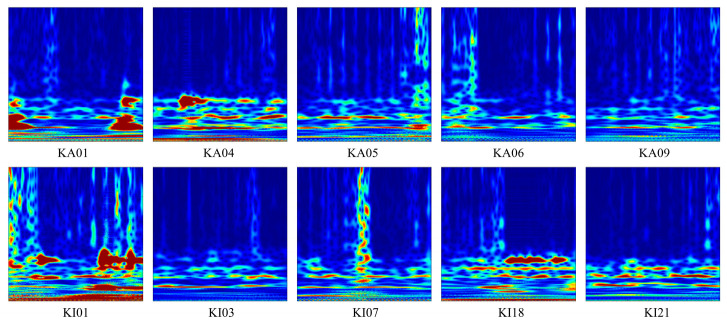
The time–frequency images by CWT.

In this paper, the CWT time–frequency images and motor current signal are used as the inputs to the model. CWT is a widely used time–frequency analysis method that provides detailed information about a signal at various frequencies and times. Compared to the other methods, CWT offers better discrimination power and resolution. Meanwhile, it is suitable for nonstationary signals, such as the impact signal and seismic signals Therefore, it is used to analyze the bearing vibration signal. However, current spectrum analysis is affected by inherent harmonics, resulting in the incomplete time–frequency analysis of the motor current signal. Therefore, the motor current signal is used directly. Each fault sample contains 1024 sampling points, with 300 samples generated for each fault type, totaling 3000 fault samples. Meanwhile, a 200-point interval is employed to increase the sample diversity. The input signals are shown in Figure 11 and Figure 12, respectively.

### 4.2. Performance Analysis of the Proposed Method

**Figure 13:**
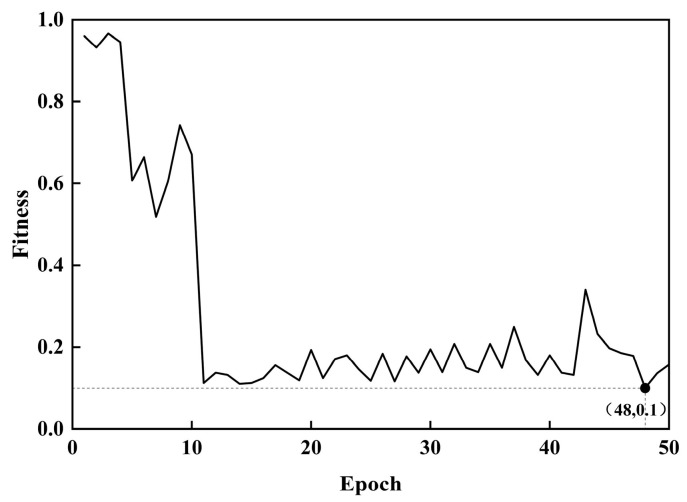
Optimized MSCNN curve with GASA.

The method presented in this study was analyzed according to the flowchart shown in Figure 9. During the pretraining and fine-tuning of the model, the loss function of the training was configured as cross-entropy, and the Adam optimization algorithm was employed for all the approaches. The initial learning rate was set to 0.001. Above all, the time–frequency graphs of the vibration and motor current signals are input into the MSCNN. Subsequently, GASA is utilized to optimize the two hyperparameters, learning rate and batch size, in the MSCNN model. The fitness curve of sample entropy during this optimization process is shown in Figure 13.

**Figure 14:**
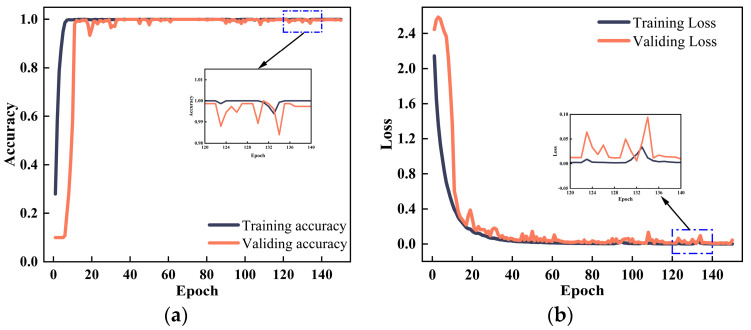
(**a**) The accuracy of the training set and validation set; (**b**) the loss values of the training set and validation set.

It can be seen from Figure 13 that the minimum sample entropy of 0.1 appears after 48 iterations, and the optimal parameter combination is (0.0006, 24). The optimized parameters are then used to train the network model, and the training results are recorded. The training results of the proposed method are shown in Figure 14. It can be seen from Figure 14 that the GASA-MSCNN converges very quickly. After about 10 training sessions, the fault diagnosis accuracy curve gradually reaches 1, but the training and verification loss are still decreasing. Around training session 40, the accuracy and loss of the model training and verification reach a steady state, with the loss value of the model gradually tending to 0.

**Figure 15:**
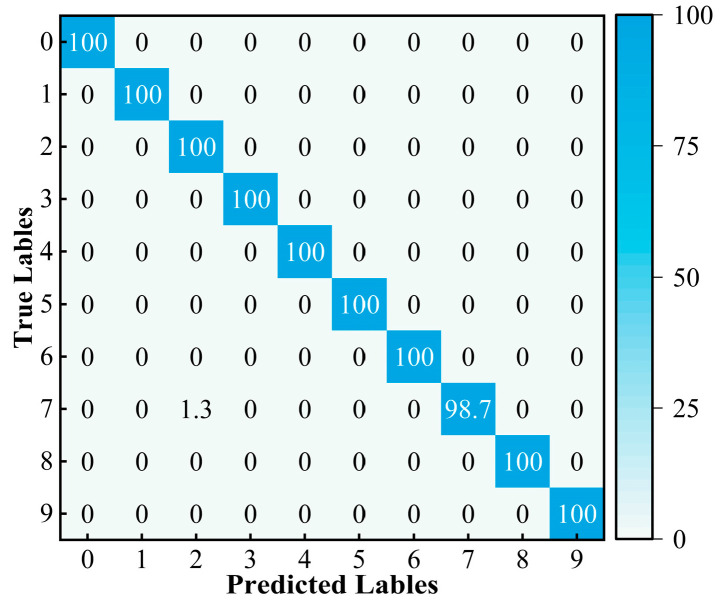
Confusion matrix of test results.

**Figure 16:**
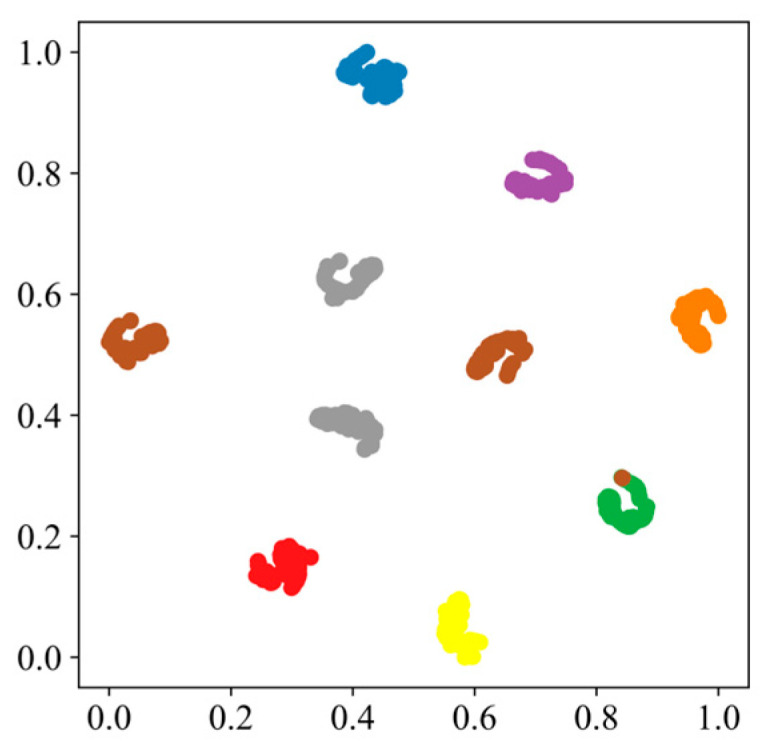
Visual clustering diagram of test results.

The diagnostic results of the validation data are shown in Figure 15. As shown in the confusion matrix in Figure 15, the overall accuracy is relatively high. For some fault types, the recognition rate reaches 100%, but there are also some errors. For example, label 7 is misclassified as label 2, identifying the inner ring electric engraving machine level 2 fault as the outer ring electric engraving machine level 1 fault. The reason for this phenomenon may be that the weight distribution in the GASA-MSCNN cannot sufficiently distinguish their features, leading to classification errors. To further demonstrate the effectiveness of the GASA-MSCNN, Figure 16 shows the T-SNE visualization of the final output results. The results show that the data points of the same type gradually cluster in the final classification. Except for some overlapping regions, the remaining fault types are completely separated, achieving good classification results. These results are consistent with the confusion matrix and establish accurate mapping relationships between the defect types and features. This demonstrates that the method performs well after training and proves that the GASA-MSCNN can achieve a strong diagnostic performance regarding rolling bearings.

To further verify the superiority of the GASA algorithm in parameter optimization, the diagnostic results of the selection process without GASA are compared. Only two variables were considered: a fixed learning rate and batch size. We selected experimental results with learning rates ranging from 0.001 to 0.0001 and batch sizes ranging from 8 to 128. The experimental results are shown in Table 3.

The table shows that the GASA algorithm greatly improves the diagnosis accuracy of the model. When the batch size is 8 and the learning rate is 0.0001, the maximum diagnostic accuracy of the model is 96.18%. When the batch size is 16 and the learning rate is 0.0006, the maximum diagnostic accuracy of the model is 95.1%. The other diagnostic results are also lower than these accuracies. Compared with the GASA-MSCNN, the accuracy of these models is reduced and the hyperparameters need to be determined empirically and through a large number of experiments. Therefore, we can conclude that the proposed method can achieve effective diagnostic accuracy.

### 4.3. Diagnosis Results under Different Noise Levels

To verify the model’s fault diagnosis superiority in variable noise circumstances, this section compares the diagnostic accuracy of the GASA-MSCNN proposed in this paper under different noise environments. The original signal is combined with Gaussian white noise to create composite signals with different signal-to-noise ratios (SNRs). The expression of the simulated signal is defined as follows:

(8) $$SNR_{db}=10\log10\left(\frac{P_{n}}{P_{s}}\right)$$

In the formula, *P_s_* and *P_n_* represent the original signal and the noise signal, respectively. The noise signal includes −4db, −2db, 0db, 2db, and 4db.

**Figure 17:**
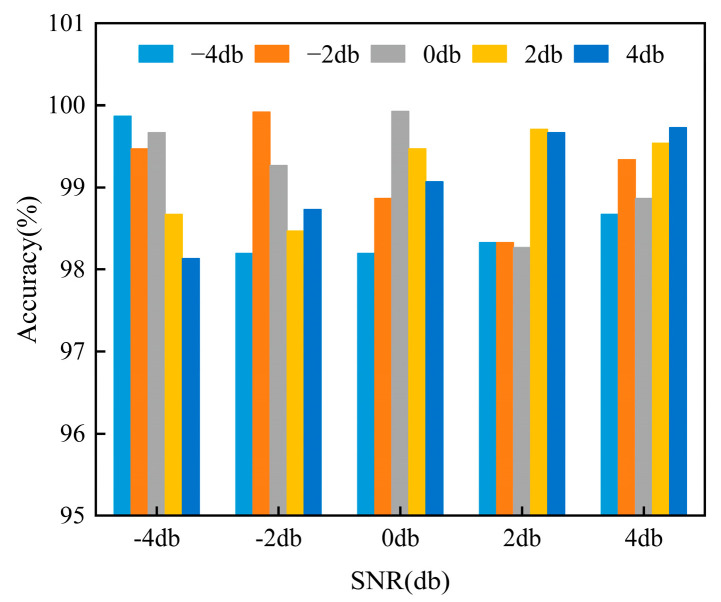
Identification results of different SNRs.

The experimental results under variable noise environments are depicted in Figure 17. Apparently, the diagnostic performance of the proposed method is above 98% under different SNRs. Specifically, the fault diagnosis accuracy is 99.87% and 99.92% in strong noise environments with SNRs of −4 dB and −2 dB, respectively. This indicates that the proposed method maintains high fault diagnosis capability even in strong noise environments. For example, in the −2 dB noise environment, the lowest diagnostic accuracy is 98.2% when −4 dB noise is the target domain, and the highest diagnostic accuracy is 99.92% when −2 dB noise is the target domain. The accuracy difference between the two diagnostic results is 1.72%, and the average diagnostic accuracy is 98.92%, indicating that the proposed method still has the ability to identify fault conditions even under noise.

**Figure 18:**
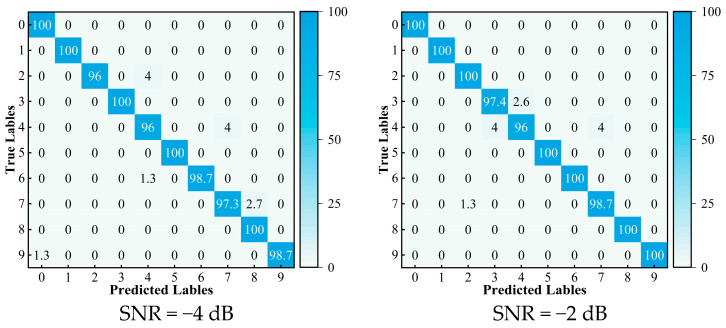

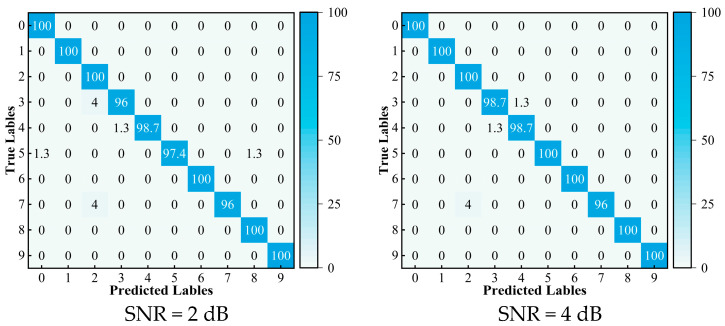
Visualization results of confusion matrices.

In order to further illustrate the effectiveness and the feature learning capability of the method under various SNRs, the confusion matrices are generated utilizing the diagnosis results for −4 dB, −2 dB, 2 dB, and 4 dB. The results are depicted in Figure 18. Here, the horizontal axis represents the predicted label, and the vertical axis represents the actual label. For instance, in a −2 dB noise environment, the diagnostic accuracy exceeds 98% for all the categories except 3 and 4. Specifically, the accuracy for category 3 is 97.4%, for category 4 is 96%, for category 7 is 98.7%, and for the other categories is 100%. In summary, the confusion matrix visualization further demonstrates the high diagnostic accuracy of the GASA-MSCNN model across various categories and provides more reliable results.

### 4.4. Comparative Experiments among Different Methods

**Figure 19:**
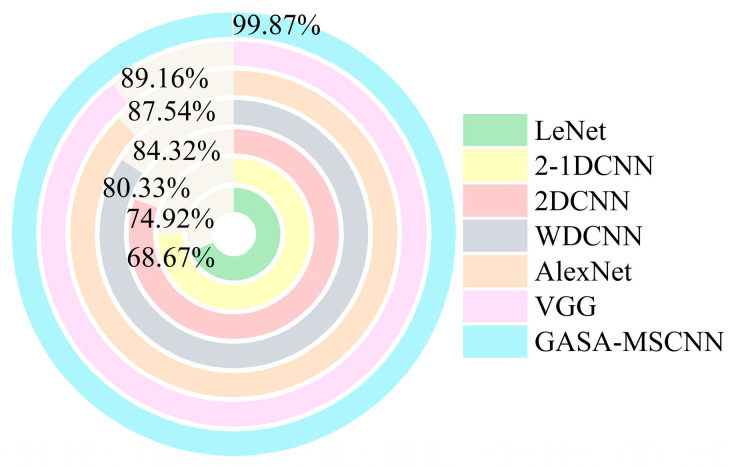
Comparison of recognition accuracy between the different models.

**Figure 20:**
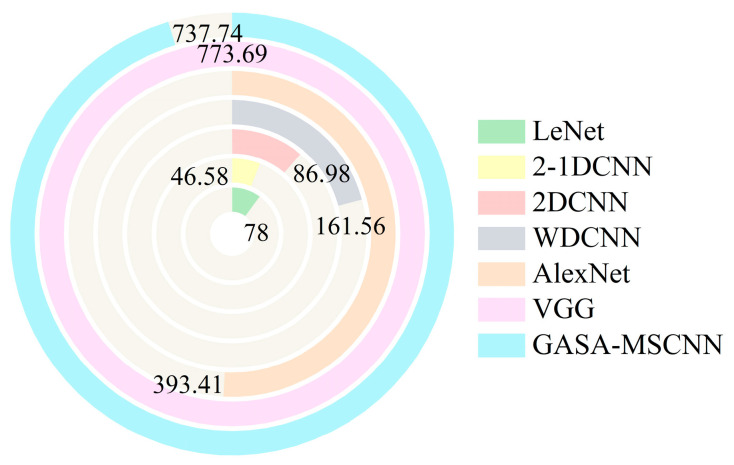
Comparison of recognition times between the different models.

To further verify the performance of the proposed method, it was compared with several widely used methods (VGG, LeNet, AlexNet, etc.) under the same conditions as GASA-MSCNN. These methods include those that use CWT time–frequency images alone, those that rely solely on vibration signals, and those that combine both vibration and current signals. The accuracy and training time of the experiments are summarized in Figure 19 and Figure 20, respectively. In terms of the diagnostic results, LeNet had the lowest accuracy at 68.6%, followed by 2-1DCNN at 74.92%. The diagnostic accuracies of AlexNet and VGG were 87.54% and 89.16%, respectively. However, compared to the GASA-MSCNN model, the diagnostic accuracies of these methods are still lower than 99.87%. Among the comparison models, VGG achieved the highest diagnostic accuracy but also had the longest diagnostic time, approximately 773.69 s. Moreover, 2-1DCNN had the shortest diagnostic time, 46.58 s, but its accuracy was only 74.92%. Although the proposed method has a relatively long training time, it achieves high diagnostic accuracy. This demonstrates that the proposed method offers the highest detection accuracy and is clearly superior to the other diagnostic methods.

## 5. Conclusions

In order to make full use of the correlation and complementarity between multi-source data and avoid the problem of manually setting the network model hyperparameters, this paper proposes a novel intelligent fault diagnosis method for bearings with multi-source data and improved GASA. This method not only fuses the multi-source data but also utilizes the GASA optimization algorithm to optimize the model hyperparameters, circumventing the need for numerous experiments and manual adjustments. Concurrently, multi-scale convolution is employed to extract multi-dimensional spatial correlation information, preventing the inaccurate or incomplete localization of the impact features due to the limitations of a single-size convolution kernel. The experimental results demonstrate that the proposed method can efficaciously diagnose faults in rolling bearings and significantly reduce fault misdiagnosis. Furthermore, the superiority and robustness of the model are validated by contrasting it under different noise environments. Compared with the existing classical models, this method shows higher fault diagnosis accuracy. This indicates that the GASA-MSCNN model possesses theoretical and practical engineering significance. However, it also identifies some shortcomings. While the fault diagnosis accuracy of the proposed model is high, this also increases the training time of the model, necessitating more sophisticated hardware to achieve the desired diagnostic results in practical applications. Consequently, future research will concentrate on reducing the response time of the model in order to facilitate more rapid and precise fault diagnosis.

## Figures and Tables

**Table 1:** Working condition information of PU dataset.

Number	Rotation Speed (rpm)	Radial Force (N)	Load Torque (N/m)	Working Condition
0	1500	1000	0.7	N_15_M07_F10
1	900	1000	0.7	N_09_M07_F10
2	1500	1000	0.1	N_15_M01_F10
3	1500	400	0.7	N_15_M07_F14

**Table 2:** PU dataset working status information.

Bearing Number	Damage	Location	Damage Level	Label
KA01	EDM	OR	1	0
KA04	Fatigue: pitting	OR	1	1
KA05	Electric Engraver	OR	1	2
KA06	Electric Engraver	OR	2	3
KA09	Drilled	OR	2	4
KI01	EDM	IR	1	5
KI03	Electric Engraver	IR	1	6
KI07	Electric Engraver	IR	2	7
KI18	Fatigue: pitting	IR	2	8
KI21	Fatigue: pitting	IR	1	9

**Table 3:** Identification results of the proposed model without GASA.

Batch_Size	Learning Rate					
	0.001	0.0001	0.0002	0.0004	0.0006	0.0008
8	93.71%	96.18%	91.59%	94.99%	95.08%	95.72%
16	93.73%	90.69%	95.09%	93.62%	95.1%	92.8%
32	89.63%	86.18%	90.85%	92.26%	91.52%	90.7%
64	86.95%	75.37%	83.38%	84.15%	83.62%	87.22%
128	72.53%	56.01%	64.85%	66.77%	70.48%	72.38%

## Data Availability

All data that support the findings of this study are included within the article.
